# Cervical spinal abscess: an insidious presentation and unusual pathology

**DOI:** 10.1308/003588412X13373405384693

**Published:** 2012-09

**Authors:** A Khoriati, J Kitson, RS Deol

**Affiliations:** East and North Hertfordshire NHS Trust,UK

**Keywords:** Spinal, Abscess, Radiology

## Abstract

Spinal abscess is a rare condition. Its presentation can often be subtle and insidious. This report describes the diagnosis and management of an 87-year-old man who presented to our orthopaedic clinic. We would like to emphasise the importance of rapid diagnosis and prompt treatment in such cases.

## Case history

An 87-year-old man presented to his general practitioner (GP) with neck pain that had been worsening for several weeks. He was generally well but had been treated with a course of antibiotics for a urinary tract infection approximately one week before the onset of his symptoms. His GP arranged plain x-rays of the cervical spine, which were performed three weeks after the initial presentation. These were initially reported to have shown little other than osteoarthritic change by the hospital radiologist. By this time his symptoms had intensified to the point that he was unable to rotate his neck. A combination of opiates, anti-inflammatories and amitriptyline had been of little benefit so the patient was referred for an orthopaedic opinion.

**Figure 1:**
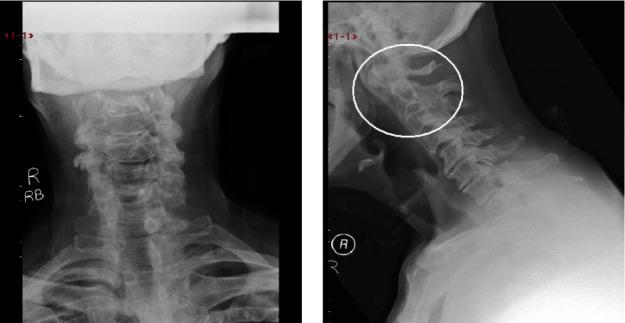
Plain x-rays of the cervical spine revealing spondylosis and increase in the distance between anterior atlas and odontoid peg

**Figure 2:**
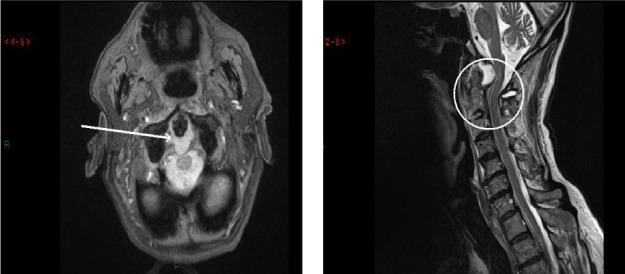
Magnetic resonance imaging: The spinal cord appears to be compressed by a collection of fluid located behind the odontoid peg.

Severe neck stiffness was noted but in the absence of any neurological deficit. Closer scrutiny of the x-rays revealed cervical spondylosis as well as an increase in the distance between the anterior atlas and the odontoid peg (Fig 1). Urgent investigation including magnetic resonance imaging (MRI) of the cervical spine and inflammatory markers was arranged. Over the next week, the patient’s condition deteriorated significantly with an unsteady myelopathic gait, spinal tenderness and a marked cervical kyphosis. MRI revealed anterior compression of the upper cervical cord by a large fluid collection behind the odontoid peg (Fig 2).**Full blood count was normal but, together with a markedly raised erythrocyte sedimentation rate of 91mm/hr, this was consistent with a diagnosis of an epidural abscess.

**Figure 3:**
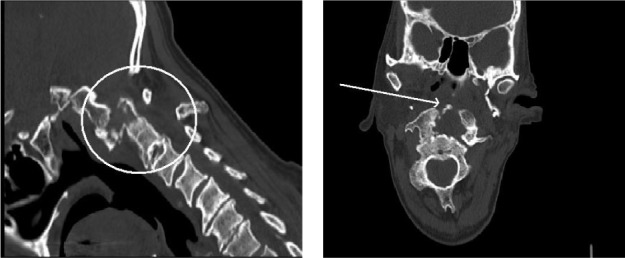
The bony destruction was clearly evident on the computed tomography and is particularly well illustrated on the sagittal views.

The patient was admitted to hospital immediately and further imaging was performed with computed tomography (CT). This showed that atlantoaxial instability had developed due to bony destruction and fracture of the odontoid peg, with an increase in the atlanto–dens interval and a corresponding decrease in spinal canal diameter (Fig 3).

**Figure 4:**
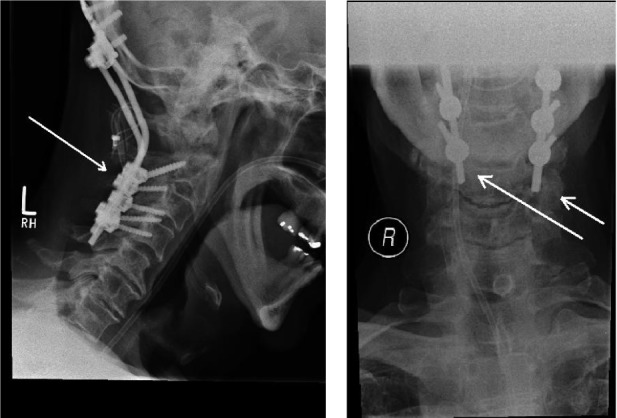
X-rays after spinal fusion

The patient was transferred urgently for specialised intervention and he underwent posterior spinal decompression and instrumented occipitocervical fusion (Fig 4), also needing a temporary tracheostomy. He made a satisfactory recovery, mobilising independently with normal gait, but required a prolonged period of rehabilitation.

## Discussion

Spinal abscess (otherwise known as epidural abscess) is a rare condition accounting for less than 1 in 3,000 hospital admissions although the incidence appears to have been increasing over the past 25 years. A number of explanations have been put forward for this including an ageing population as well as the rise of intravenous drug use.^1^ Approximately 70% of those who develop spinal abscesses are between the ages of 31 and 70.

Although the pain associated with a spinal abscess typically becomes severe and unrelenting, the presentation of this condition can be subtle and insidious. Spinal stiffness and tenderness are key clinical findings. Tenderness is the most common symptom, affecting 85% of patients with a spinal abscess.^2^ A gradual onset of vague neurological symptoms may develop, leading to sudden deterioration of motor function. Approximately 50% of spinal abscesses are either misdiagnosed or diagnosed at a very late stage.^3^

Spinal abscesses are thought to form because of bacterial infiltration into the retrovertebral epidural venous plexus. The causative organism in such cases is typically *Staphylococcus aureus* although other possible pathogens include *Escherichia coli*, *Pseudomonas*, *Brucella* and *Mycobacterium* tuberculosis.^4^

There are three key points highlighted by the above case. The first is the insidious presentation. The patient presented initially with mild neck pain, not an uncommon complaint in the elderly. The prevalence of neck pain in the adult European population is between 13% and 39%.^5^ This increases with age (up to 59% in patients over 65 years). Spinal abscesses are commonly associated with severe morbidity and mortality, paraplegia and even death being potential consequences. The mortality rate has been reported to be as high as 34%.^2^ The learning point from this case is the need for early diagnosis and urgent intervention.

Our case also demonstrates the value of the full range of imaging modalities. The changes on the initial x-rays were subtle and did not point to a specific diagnosis. While MRI revealed the fluid collection and soft tissue change, it was CT that subsequently showed the full extent of bony destruction, allowing appropriate planning for the operative management.

## Conclusions

We would like to highlight the need for a high awareness of this condition. Spinal abscesses typically develop from one of three causes. Spread may be haematogenous, from a distant infective focus, as in this case. There may be direct extension into the canal from an adjacent source of infection such as discitis. The cause can also be iatrogenic, for example from spinal injections. A thorough history and clinical examination holds the key to detecting this subtly presenting condition at an earlier stage, leading to appropriate investigations without delay.
